# Cognitive function at 2443 μmol/l creatinine

**DOI:** 10.1186/1471-2369-13-86

**Published:** 2012-08-15

**Authors:** Sabrina Schneider, Anne-Kathrin Malecki, Olaf Boenisch, Robby Schönfeld, Jan T Kielstein

**Affiliations:** 1 Department of Psychology, Martin-Luther University, Halle-Wittenberg, Germany; 2 Department of Nephrology and Hypertension, Medical School Hannover, Hanover, Germany

**Keywords:** Uremia, Trail making A, Executive function

## Abstract

**Background:**

One hallmark of uremia is the impairment of neuro-cognitive function. Anecdotal clinical description from the early days of chronic dialysis therapy impressively illustrates the improvement of those functions by chronic hemodialysis treatment. Fortunately, today, uremia is only rarely observed in industrialized countries as many patients seek medical/nephrological attention prior to the occurrence of deadly complications of uremia.

**Case presentation:**

We report a rare case of severe uremia and describe the day to day improvement in neuro-cognitive function by dialysis using state of the arte test battery – starting at a serum creatinine of 2443 μmol/l.

**Conclusions:**

Especially executive functions, which are assumed to be localized in the frontal cerebral regions, are impaired in severe uremia and improve remarkably with the correction of severe uremia, i.e. initiation of dialysis.

## Background

In 1847 Piorry coined the term “uremia” describing a multi-facetted clinical syndrome consisting of digestive and neurological abnormalities secondary to renal failure and the resulting endogenous intoxication [1]. One well documented finding in patients with chronic kidney disease (CKD) is the impairment of cognitive function. Kurella et al. could show that decreased eGFR (estimated glomerular filtration rate) in postmenopausal woman is associated significantly with impairment in global cognition, executive function, language, and memory [2]. In a community-based cross-sectional study Elias et al. found that global performance and specific cognitive functions are negatively affected early in CKD [3]. Interestingly not only a decrease in GFR but also elevated urinary albumin/creatinine ratios is independently associated with faster decline in cognitive function [4]. Moreover, moderate renal impairment, has been shown to be associated with an excess risk of incident dementia among individuals in good to excellent health [5]. The pathophysiological mechanism for this is still not clearly understood and range from urea to the endogenous NOS inhibitor ADMA [6,7]. Moreover, there are no simple screening tools to diagnose cognitive function in CKD5D patients [8] While there is no doubt that severe uremia impairs cognitive function, the effect of a single dialysis session or dialysis intensity on cognitive impairment is controversially discussed. One study could not find any effect of a single dialysis on insensitive test like minimental state examination [9]. Other authors reported that the removal of uremic toxins by hemodialysis lead to an improvement in cognitive processing [10]. Also the potential effect of an increase of dialysis intensity on cognitive discussion is controversially discussed. While one pilot study in 12 patients showed improved general cognitive efficiency 6 months after switching from thrice weekly to nocturnal hemodialysis [11] results from the frequent hemodialysis network nocturnal trial showed no effect of increased dialysis intensity on cognitive performance, measured by Trail Making Test B only [12]. We monitored the neuro-cognitive function of a patient with severe uremia in chronic humoral rejection secondary to non-adherence to immunosuppressive therapy after renal transplantation, starting at a serum creatinine of 2443 μmol/l.

## Case presentation

A 27-year-old pale Caucasian male was admitted into the emergency room of our tertiary care hospital with the diagnosis of uremia. Upon arrival the overweight patient (BMI 32.7 kg/m²) had a blood pressure of 164/91 mmHg and a heart rate of 110/min. He presented with nausea and diarrhea, but was alert and awake. The remainder of the physical examination showed pericardial friction rub as well as a uremic foetor. His mucus membranes were dry and pale. His past medical history was significant for a living related kidney transplantation in 2008 for chronic interstitial nephritis and reflux nephropathy.

About ten weeks prior to the admission he stopped taking the immunosuppressive medication which consisted of tacrolimus, prednisolone and mycophenolat-mofetil. The main reason for discontinuation was allegedly his financial situation after a job loss, which did not allow him to pay the necessary co-payment for the immunosuppressive drugs. He did neither seek help from the hospital nor from the welfare authorities/social services. Laboratory analysis was remarkable for a serum creatinine of 2443 μmol/l a serum urea of 67.6 mmol/l and a serum phosphorous level of 2.69 mmol/l. His venous blood gas analysis showed a metabolic acidosis (pH 7.27, bicarbonate 14 mmol/l). He was anemic with a hemoglobin level of 6.9 g/dl.) Renal biopsy showed a severe irreversible damage of the graft due to chronic humural rejection. After transfer to our unit hemodialysis was initiated. The patient also received two units of packed red blood cells, which lead to an increase in hemoglobin level to 8.8 g/dl. Peak creatinine declined under the initiation of dialysis. Due to an ongoing research project on the evaluation of cognitive function in dialysis patients we were able to use a battery of established test for neuro-cognitive function for frequent monitoring. The patient consented to take part in frequent psychological tests consisting of computer-based attention tasks, memory tasks (short-term and long-term memory) and also several tasks to examine executive functions. The full test battery consisted of Wechsler Digit Span, Stories of Rivermead Behavioral Memory Test (RBMT), TMT A and B, Alertness and Go/NoGo subtest of *Testbatterie zur Aufmerksamkeitsprüfung* (TAP), Key Search and Zoo Map of Behavioral Assessment of the Dysexecutive Syndrome (BADS), *Regensburger Wortflüssigkeitstest* (RWT) as well as Rey Complex Figures.

The neuropsychological assessments were conducted by trained personal in a quiet environment. Tests were all performed between 08:00 and 10:00 AM to minimize the effect of circadian changes. To retain and optimize the patients´ compliance and motivation as well as to avert effects of interference between the several domains, the tests alternate in the order related to their verbal and visuo-constructive characteristic. Initial practice trails were administered for the following tests (TMT A, TMT B and TAP) to make sure that the task was understood by the patient. Tests with known training effects were not regularly repeated. The test battery with no or minimal influence by learning effects consisted of Wechsler Digit Span, Stories of RBMT, TMT A and B, Alertness and Go/NoGo subtest of TAP as well as Rey Complex Figures [13].

**Figure 1:**
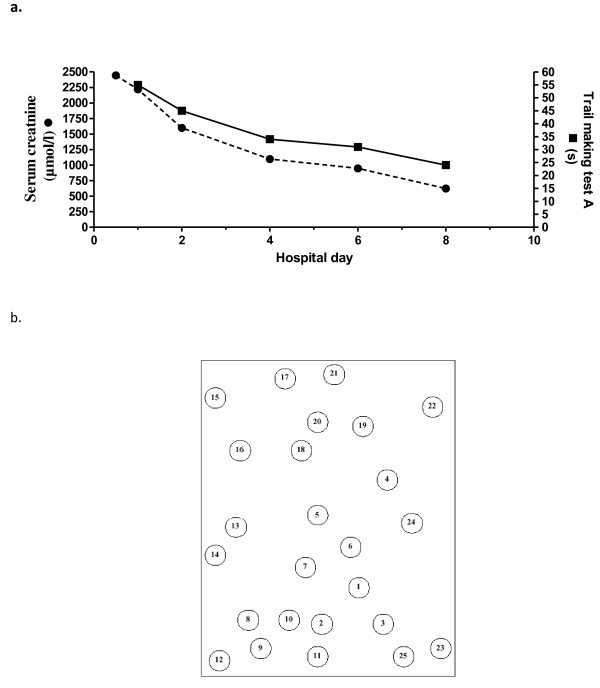
**Reduction of creatinine by dialysis and concomitant improvement of the trail making test A in a uremic patient.** (Figure 1a) In trail making test A 25 circles are distributed over a sheet of paper, numbered 1 to 25 (Figure 1b). The patient should draw lines to connect the numbers in ascending order as fast as possible.

**Figure 2:**
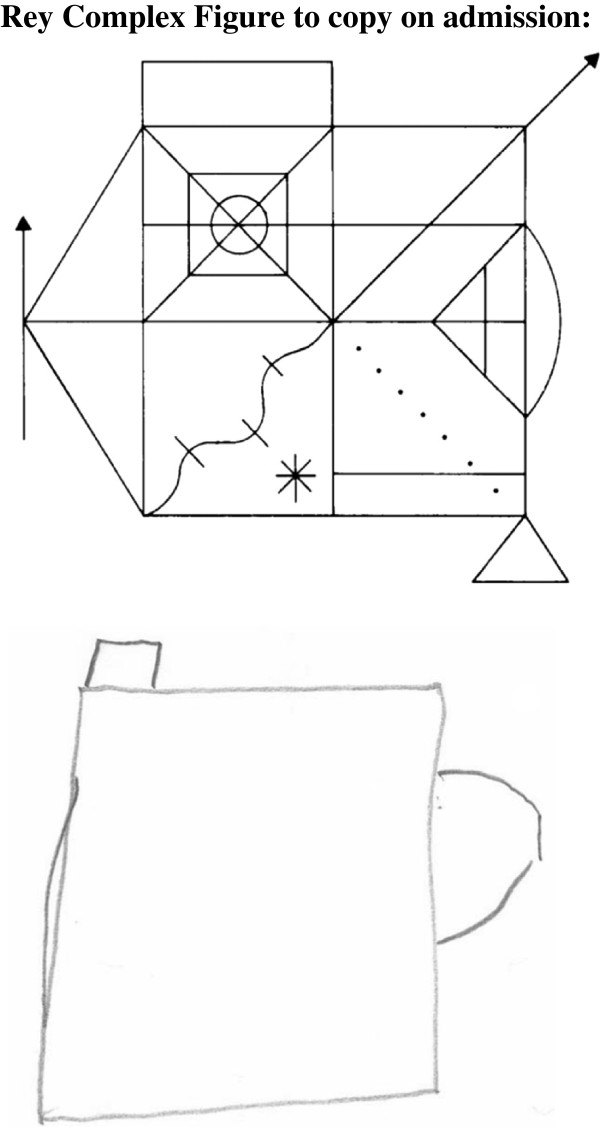
**Rey Complex Figure to copy on admission.** Recall (after three minutes) at baseline during uremia.

**Figure 3:**
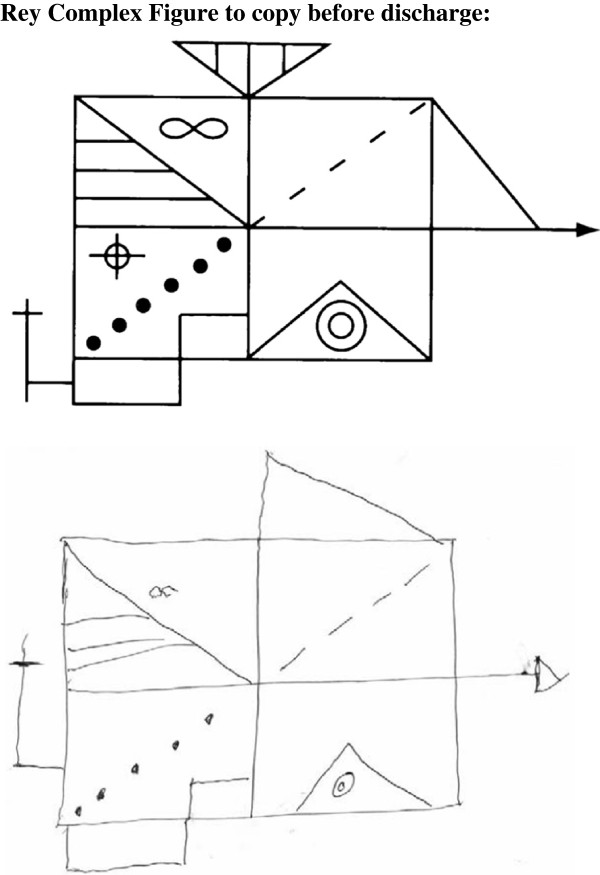
**Rey Complex Figure to copy before discharge.** Recall **(**after three minutes) after the fourth dialysis sessions.

In parallel to the correction of uremia, indicated by the marker substances creatinine, urea we saw general improvement of cognitive function (Figure 1a, 2, 3). However, after the first hemodialysis the patient showed the lowest performance, possibly due to a slight disequilibrium syndrome despite the fact that the first hemodialysis lasted only 120 minutes. The disequilibrium syndrome is caused by rapid removal urea resulting in an increased osmolality in the brain tissue (as compared to the serum) leading to water flux in the brain. i.e. cerebral edema. Indeed, in our patient there was a small but steady decrease in the calculated serum osmolality **(**Table 1)**.** The neuropsychological performance is reported in the Table 1. Scores on Wechsler Digit Span (forward) increased with ongoing testing. Further verbal memory performance, tested with Stories of RBMT, also increased. But in RBMT we found a performance loss on the second testing. All in all the short-term memory performance (on Wechsler Digit Span and RBMT) improved on the last testing compared with the first testing. Figural memory performance in Rey Complex Figures was consistently in an average range, (Table 1 and Figure 3), except for the second testing, were we found a marked decrease in performance (Figure 2). Patient´s memory span plus standardized scores of verbal and figural memory showed no evidence of neuropsychological deficits for the patient compared to established control ranges. Interestingly, in the computer-based *alertness* attention task, we also found an improvement concomitant with the improvement of uremia. The assessment of executive functions after admission showed a marked underperformance even after correcting for the patients´ age. Concomitant with the initiation of dialysis therapy the patient considerably improved. Performance on the TMT A increased in the first three tests (Figure 1). During the whole test period we observed an improvement from underperformance to average performance. Accordingly to TMT B we found a salient increase not until the fourth testing. TMT A and B performance was perfectly correlated with the correction of the serum creatinine level (Spearman's rank correlation: rs=1.0, p <.05). Nevertheless the performance on TMT B was clearly below-average, thereby indicating a continuous deficit in cognitive function, which is only “average” in the last of the all tests. The correction of uremia only slightly improved verbal fluency tasks (RWT).

**Table 1:** Raw scores and percentile of neuropsychological test battery

	**RAW SCORES (PERCENTILE)**					
	**test # 1**	**test # 2**	**test # 3**	**test # 4**	**test # 5**	**test # 6**
**Hospital day # TESTS**	**1**	**2**	**4**	**5**	**7**	**8**
**Story RBMT (words)**						
recall	**8**	**5.5**	**10**	**11**	**11.5**	**13**
**Rey Complex Figure**						
Immediate and delay	**41(58–62)**	**13 (10)**	**42.5 (62–66)**	**40 (58)**	**50 (79)**	**35 (46–50)**
**Wechsler Digit Span**						
forward	**6**	**6**	**7**	**7**	**7**	**9**
backward	**5**	**4**	**6**	**6**	**6**	**6**
**Trail Making Test (seconds)**						
A	**55 (<10)**	**45 (<10)**	**34 (10–20)**	**35 (10–20)**	**31 (20–30)**	**24 (40–50)**
B	**129 (<10)**	**109 (<10)**	**101 (<10)**	**74 (<10)**	**73 (<10)**	**64 (20)**
B/A	**2.3**	**2.4**	**3.0**	**2.1**	**2.4**	**2.7**
**TAP (milliseconds)**						
**Alertness**						
with acoustic signal	**260 ± 57 (24)**	**226 ± 47 (38)**	**216 ± 30 (38)**	**273 ± 40 (12–14)**	**208 ± 27 (50)**	**200 ± 31 (73)**
without acustic signal	**279 ± 49 (10)**	**241 ± 34 (31)**	**233 ± 29 (38)**	**257 ± 39 (27)**	**225 ± 38(58)**	**207 ± 23 (73)**
**RWT (words)**						
lexical	**15 (50–75)**	**17 (50)**				**20 (84)**
lexical-change	**15 (10–25)**	**15 (16–25)**				**15 (10–25)**
semantic	**22 (<10)**	**23 (16)**				**26 (25–50)**
semantic-change	**26 (>90)**	**16 (23)**				**28 (>90)**
**Laboratory parameters Serum Creatinin (μmol/l)**	**2443**	**1599**	**1097**		**948**	**629**
**Serum Urea (mmol/l)**	**67.6**	**41.6**	**32.7**		**29.9**	**17.8**
**Phosphorus (μmol/l)**	**2.69**	**2.21**	**2.07**		**./.**	**./.**
**Sodium (mmol/l)**	**138**	**139**	**139**		**136**	**139**
**Calculated osmolality (mosmol/l)**	**348**	**336**	**325**		**316**	**311**
**Hemoglobin (g/dl)**	**6.3**	**8.8**	**7.6**		**7.8**	**8.7**

## Conlcusions

The first report on the treatment of chronic uremia by intermittent hemodialysis by Scriber in 1960 identified already the main problems in treating CKD 5 D patients [12]. Aside from anemia, sodium and fluid overload as well as hypertension Scribner et al. describe *“increased fatigability, muscle cramps, irritability and lethargy”* as symptoms of uremia. Sufficient intensity of dialysis in the first two chronic hemodialysis patients lead to a situation in which *“neither patient has yet shown the relentless loss of weight and the mental deterioration which has been encountered in the past when less intensive dialysis therapy was employed”*. This description epitomizes that at least some of the alterations in cognitive function in uremia are reversible in nature and could be due to the accumulation of uremic toxins. To our knowledge we report for the first time a sequential examination of a patient in severe uremia using an extensive state of the art battery of neurocognitive tests. A widespread armentarium has previously only been employed in a cross-sectional study of chronic hemodialysis patients [13]. A study comparing the effect of acute removal of uremic toxins has to our knowledge so far not been conducted. Bae and Park compared Contingent Negative Variation (CNV) recorded with electroencephalogram 1 h before and 1 h after a dialysis session finding no significant difference [14]. The neuropsychological tests (MMSE and TMT) they used were only for a cross-sectional analysis and not to compare pre- and post- dialysis cognitive status. Williams et al. studied patients at intervals of 1, 24, and 67 hours after the last weekly hemodialysis session and found fluctuations in memory and attention with a time dependent increase in impairment after the dialysis session [15].

The very nature of a case report does not allow generalizing the results we obtained in a peculiar case of uremia. Nonetheless we conclude that especially executive functions, which are assumed to be localized in the frontal cerebral regions, are impaired in severe uremia and improve remarkably with the initiation of dialysis treatment.

## Consent

Written consent was obtained from the patient for publication of this study. Ethical approval was for reporting this case was obtained from the Medical School Hannover.

## Competing interests

The authors declare that they have no competing interests.

## Authors’ contributions

SS and AM conducted the neurocognitive testing. OB and JTK were the treating physicians of the patient reported. SS, JTK and RS evaluated the test results. All of the authors have participated in the discussion and in writing of the submitted manuscript. All authors read and approved the final manuscript.

## Pre-publication history

The pre-publication history for this paper can be accessed here:

http://www.biomedcentral.com/1471-2369/13/86/prepub
